# Cerebrospinal fluid neopterin as a biomarker of neuroinflammatory diseases

**DOI:** 10.1038/s41598-020-75500-z

**Published:** 2020-10-26

**Authors:** Marta Molero-Luis, Didac Casas-Alba, Gabriela Orellana, Aida Ormazabal, Cristina Sierra, Clara Oliva, Anna Valls, Jesus Velasco, Cristian Launes, Daniel Cuadras, Belén Pérez-Dueñas, Iolanda Jordan, Francisco J. Cambra, Juan D. Ortigoza-Escobar, Carmen Muñoz-Almagro, Angels Garcia-Cazorla, Thais Armangué, Rafael Artuch

**Affiliations:** 1 Institut de Recerca Sant Joan de Déu, Barcelona, Spain; 2 Clinical Biochemistry Department, Hospital Sant Joan de Déu, Passeig Sant Jan de Déu, 2, Esplugues de Llobregat, 08950 Barcelona, Spain; 3 Pediatric Neurology Department, Hospital Sant Joan de Déu, Barcelona, Spain; 4 Pediatrics Department, Hospital Sant Joan de Déu, Barcelona, Spain; 5 Fundació Sant Joan de Déu, Barcelona, Spain; 6 Pediatric Neurology Research Group, Hospital Vall d’Hebron - Institut de Recerca (VHIR), Barcelona, Spain; 7 Pediatric Intensive Care Unit, Hospital Sant Joan de Déu, Barcelona, Spain; 8 Pediatric Infectious Diseases Research Group, CIBERESP, Institut Recerca Hospital Sant Joan de Déu, Barcelona, Spain; 9 Movement disorder Unit ERN-RND, Hospital Sant Joan de Deu, Barcelona, Spain; 10 CIBERER-Instituto de Salud Carlos III, Barcelona, Spain; 11 CIBER de Epidemiología y Salud Pública (CIBERESP), ISCIII, Barcelona, Spain; 12 Department of Medicine, Universitat Internacional de Catalunya, Barcelona, Spain; 13 Neuroimmunology Program, Institut d’Investigació Biomèdica August Pi i Sunyer (IDIBAPS)-Hospital Clínic, University of Barcelona, Barcelona, Spain; 14 Pediatric Neuroinmunology Unit, Sant Joan de Deu Children’s Hospital, University of Barcelona, Barcelona, Spain

**Keywords:** Biomarkers, Neurology

## Abstract

The elevation of neopterin in cerebrospinal fluid (CSF) has been reported in several neuroinflammatory disorders. However, it is not expected that neopterin alone can discriminate among different neuroinflammatory etiologies. We conducted an observational retrospective and case–control study to analyze the CSF biomarkers neopterin, total proteins, and leukocytes in a large cohort of pediatric patients with neuroinflammatory disorders. CSF samples from 277 subjects were included and classified into four groups: Viral meningoencephalitis, bacterial meningitis, acquired immune-mediated disorders, and patients with no-immune diseases (control group). CSF neopterin was analyzed with high-performance liquid chromatography. Microbiological diagnosis included bacterial CSF cultures and several specific real-time polymerase chain reactions. Molecular testing for multiple respiratory pathogens was also included. Antibodies against neuronal and glial proteins were tested. Canonical discriminant analysis of the three biomarkers was conducted to establish the best discriminant functions for the classification of the different clinical groups. Model validation was done by biomarker analyses in a new cohort of 95 pediatric patients. CSF neopterin displayed the highest values in the viral and bacterial infection groups. By applying canonical discriminant analysis, it was possible to classify the patients into the different groups. Validation analyses displayed good results for neuropediatric patients with no-immune diseases and for viral meningitis patients, followed by the other groups. This study provides initial evidence of a more efficient approach to promote the timely classification of patients with viral and bacterial infections and acquired autoimmune disorders. Through canonical equations, we have validated a new tool that aids in the early and differential diagnosis of these neuroinflammatory conditions.

## Introduction

Tetrahydrobiopterin (BH4) is the cofactor of tyrosine (EC 1.14.16.2), tryptophan (EC 1.14.16.7), and phenylalanine hydroxylases (EC 1.14.16.1) for nitric oxide synthase (E.C.1.14.13.39) and alkylglycerol monooxygenase (EC 1.14.16.5) activities^1^. Guanosine triphosphate cyclohydrolase I (EC 3.5.4.16; GTPCH) is the rate-limiting enzyme for BH4 biosynthesis^2^. During this reaction, neopterin is released from the cells into biological fluids, representing a surrogate biomarker used to estimate the activity of this enzymatic step^2^. Under inflammatory/immune events, GTPCH activity is triggered by interferon-gamma. Hence, neopterin concentrations are higher in different biological fluids when immune-mediated and inflammatory disorders appear, in which T-helper 1 cells and macrophages are involved^3,4^.

It has been demonstrated that neopterin in the brain is independently produced, as there is no correlation between the concentrations of neopterin in the plasma and cerebrospinal fluid (CSF) of patients with immune-inflammatory disorders^5–8^. Regarding neopterin cellular sources in the brain, it has been suggested that microglia and astrocytes are candidates to produce neopterin since these cells respond to interferon-gamma^9^. Furthermore, biogenic amine-producing cells which depend on BH4 biosynthesis, could also synthesize neopterin, including dopaminergic and serotoninergic neurons. The production of neopterin has also been demonstrated in isolated peripheral neurons (dorsal root ganglia neurons) under inflammatory conditions^9^. Neopterin is not only a biomarker for immune-inflammatory disorders as, during infections, it can increase oxidative stress within the infected cells to eliminate a pathogen^4^. Additionally, neopterin acts as a cytoprotective molecule in nonimmune resident cells^9^. Other biological roles include the translocation of nuclear factor kappa B, increased intracellular calcium, increased proto-oncogene expression, apoptosis, and reduced cell viability in different human cells^9^.

The elevation of neopterin in the CSF has been reported in several disorders, including acute viral and bacterial infections^10–15^ and chronic neuroinflammatory diseases, among others^7,8,16,17^. A cut-off value of 61 nmol/L for neopterin in the CSF has been proposed to allow for discrimination between central nervous system inflammatory and non-inflammatory disorders in paediatric populations^8^. However, it is not expected that neopterin alone can discriminate among different neuroinflammatory aetiologies^8^.

## Methods

### Aim

To analyse the CSF biomarkers neopterin, total proteins, and leukocytes in a large cohort of paediatric patients with neuroinflammatory disorders, and report that when combined, these biomarkers may allow for the rapid discrimination of different neuroinflammatory diseases.

### Design and setting

This was an observational retrospective and case–control study over a period of 15 years (2004–2018).

### Patients

We retrospectively recruited CSF analysis reports from patients with a definitive diagnosis of neuroinflammatory diseases (lumbar puncture was indicated following our diagnostic clinical protocols). CSF samples were collected during the debut of the disease, and therefore no aetiological treatment was indicated at that moment. The exclusion criteria were as follows: (1) samples with traumatic (haematic) puncture and inadequate conditions for sample collection and preservation, such as a lack of light and temperature preservation and (2) CSF samples from patients with no relevant clinical information or without an aetiological diagnosis. Finally, CSF samples from 277 patients (53% males and 47% females, average age: 5.8 years; standard deviation 5.2 years; age range: 1 month–21 years) were included in the study and classified into four different groups:

- Patients with viral meningoencephalitis (n = 107) triggered by enterovirus (n = 75), herpes simplex (n = 5), Epstein–Barr (n = 2), human parainfluenza (n = 2), varicella zoster (n = 1), and measles viruses (n = 1). Patients with lymphocytic aseptic meningoencephalitis were also included (n = 21).
- Patients with bacterial meningitis (n = 15) caused by *Streptococcus pneumoniae* (n = 6), *Neisseria meningitidis* (n = 4), *Mycobacterium tuberculosis* (n = 2), and *Escherichia coli*, *Streptococcus agalactiae* and *Haemophilus* (n = 3).
- Patients with acquired immune-mediated disorders (n = 48), including 36 patients with brain immune diseases (23 acquired demyelinating syndromes, 10 autoimmune encephalitis, 2 central nervous system vasculitis, and one opsoclonus myoclonus syndrome), 10 with autoimmune diseases of the peripheral nervous system (7 Guillain–Barré syndrome and 3 chronic demyelinating inflammatory polyneuropathy), and 2 with combined central and peripheral nervous system involvement (combined Bickerstaff encephalitis and Guillain–Barré syndrome).
- Control group (n = 107). Patients where lumbar puncture was initially indicated to rule out bacterial and viral meningitis, but after clinical follow-up, this was ruled out, and the results after biochemical and microbiological studies were negative. To avoid selection bias, we also recruited all neuropaediatric patients who underwent lumbar puncture in the outpatient clinics during 2019 for aetiological diagnosis of neurometabolic diseases with no suspicion of neuroinflammatory disorders.

### Laboratory studies

CSF samples were collected by lumbar puncture as previously reported^18^. Once CSF samples were collected, they were stored at − 80 °C, protected from light until the moment of neopterin analysis. WBCs and total proteins were analysed the same day as the lumbar puncture. Neopterin was analysed with reverse-phase high-performance liquid chromatography with fluorescence detection following a previously reported procedure^18^. Briefly, to oxidize pterins to biopterin and neopterin, 150 µL of CSF was mixed with 15 µL of 1 mol/L HCL and 1 mg of manganese dioxide. After 10 min of incubation at room temperature, the mixture was filtered through a ultrafree Millipore filter by centrifugation (10 min at 12.000 × *g*, 4 °C). Then, 20 µL of the supernatant was injected onto the HPLC. The mobile phase consisted of 1 µmol/l potassium phosphate plus methanol (95/5 v/v). Excitation was 350 nm, and emission was 450 nm. Typical chromatograms of the neopterin calibrator and real CSF samples (displaying normal and high neopterin concentrations) are depicted in additional file 1. Total proteins were analyzed with standard automated spectrometric procedures and leukocytes were counted in a manual counting chamber using undiluted CSF the same day as the lumbar puncture.

### Microbiological studies

Microbiological diagnosis techniques were performed according to the clinical suspicions of the patients, including bacterial CSF cultures and several specific real-time polymerase chain reactions for DNA/RNA detection of *N. meningitidis, S. pneumoniae*, *Herpeviridiae,* and enterovirus^19–21^. Molecular testing for multiple respiratory pathogens was also included for patients with meningoencephalitis and acute respiratory infections. Since 2016, we also included the multiplex molecular assay Filmarray Meningoencephalitis panel for selected patients when the routine techniques were negative^22^.

### Antibodies against neuronal and glial proteins

Neuronal antibody testing in the CSF samples and in the paired serum samples when available was performed at the IDIBAPS-Hospital Clinic, University of Barcelona, using previously reported techniques^23–25^. In brief, to determine the presence of neuronal surface antibodies, samples (serum 1:200; CSF 1:2) were examined with the immunohistochemistry of rat brain tissue processed to detect most antibodies against neuronal cell surface proteins (NMDA, mGluR5, AMPA, GABAB, GABAA, receptors and LGI1, Caspr2, and DPPX proteins)^23^. If positive, the identity of the antigen was confirmed with the corresponding cell-based assay (CBA)^24^. Additionally, all of the samples were systematically tested for antibodies against MOG using a CBA with live HEK293 cells transfected with a full-length transcript with the C-terminal region fused to EGFP (serum diluted 1:160 and CSF 1:2)^25^.

### Statistics

Analysis of the data distribution (Kolmogorov–Smirnov test) showed a non-Gaussian distribution. The Spearman correlation test was applied to search for correlations between patient age and neopterin values. Since no correlation was observed between neopterin and age in 107 controls, a unique reference group was established (from 1 month to 21 years). We applied ROC analysis to calculate the cut-off value for the different clinical groups with respect to controls. The three continuous variables (CSF proteins, leukocytes, and neopterin) were transformed into logarithmic (log) values and ANOVA with Bonferroni correction testing was applied to search for significant differences between the patient groups for the three CSF biomarkers. Statistical significance was defined as *p* < 0.05. Statistical calculations were performed using SPSS 23.0 (IBM Corp.; Armonk, NY) and R 2.6 software (R Foundation for Statistical Computing; Vienna, Austria).

Canonical discriminant analysis of the three CSF biomarkers and the patient’s age was conducted to establish the best discriminant functions for the classification of the different clinical groups, as previously reported^26^. This statistical technique creates linear combinations of variables that best separate the different clinical groups.

A leave-one-out cross-validation assessment was performed to validate the canonical discriminant analysis results. This test predicts the single individual classification while considering the rest of the participants. Thus, a single patient will not participate in the process of his own classification. Moreover, model validation was conducted in an independent cohort of patients recruited during 2019 from hospitalized patients (n = 95; age range: 1 month–17 years, average = 6.4 years; SD = 5.6; 49.5% males, 50.5% females). Exclusion criteria were the same as previously stated. For validation purposes, these patients were classified in the same clinical groups as the present work, plus a new group of nine patients with a diagnosis of Aicardi-Goutières syndrome caused by pathogenic variants in the *RNASEH2B*, *ADAR* and *IFIH1* genes. Clinical and laboratory data of this cohort of patients are stated in additional file 2.

### Ethical approval

This study was approved by the Institutional Review Board of Hospital Sant Joan de Déu (IRB number ART-14-19). Samples were taken in accordance with the 2013 revised Helsinki Declaration of 1964. Parents of patients signed informed consent for diagnostic interventions.

## Results

**Figure 1:**
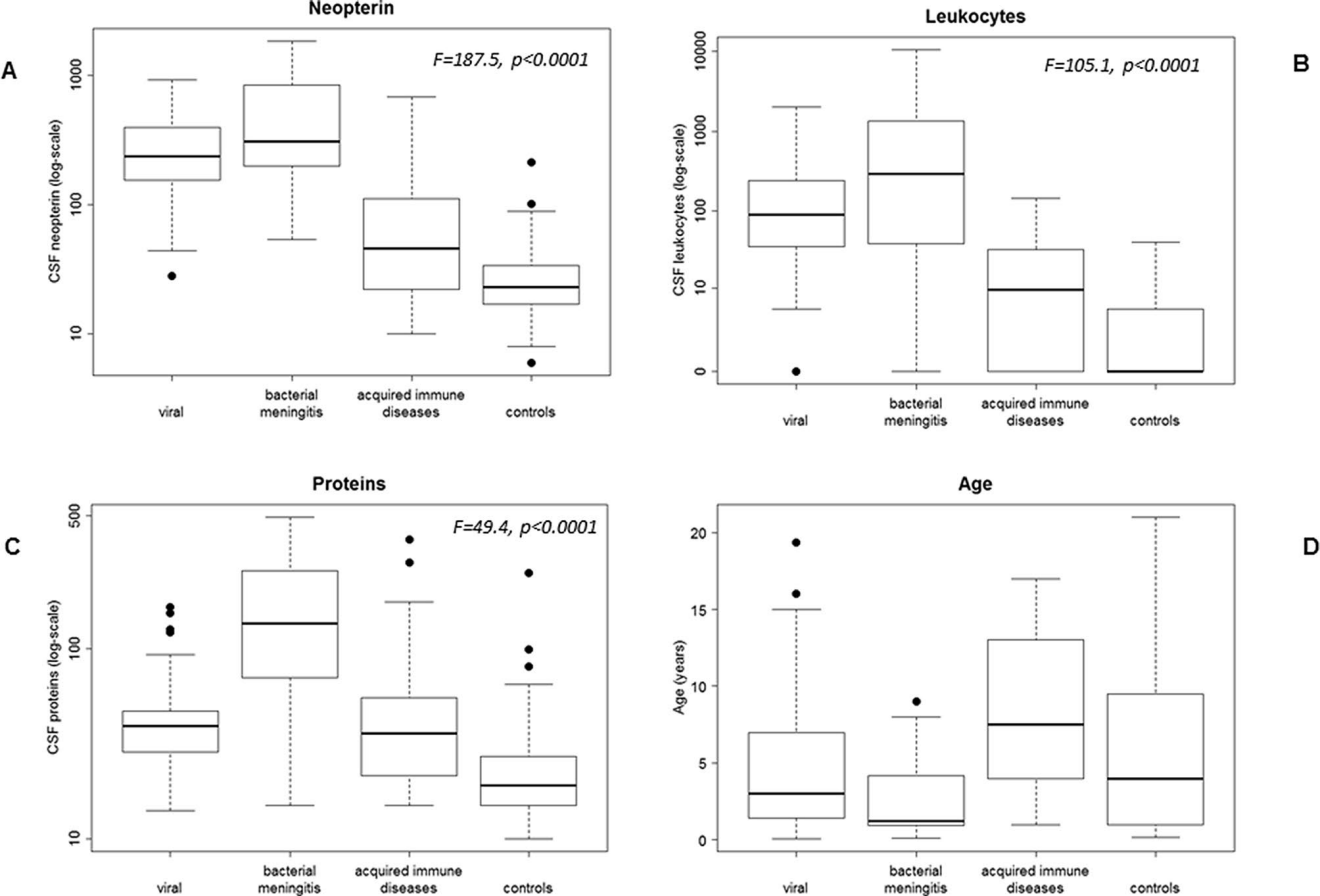
Box plot of log-transformed variables. (**A**) CSF neopterin values. (**B**) CSF leukocyte count. (**C**) CSF total protein values. (**D**) Patient’s age. Regarding neopterin and leukocytes, the highest values were observed in the bacterial and viral meningitis groups and showed significant differences when compared with the other groups (ANOVA with Bonferroni correction). For CSF proteins, the highest values were detected in the bacterial infection group, which showed significant differences when compared with all of the other groups. Finally, the acquired autoimmune group showed the highest values with regard to the patient’s age variable. The individual statistical differences among groups are stated in additional file 4. The length of the boxes indicates the interquartile space (p25–p75); the horizontal line into the box represents the median (p50); and the circles indicate outlier values.

The CSF biochemical data of the patient groups are presented in Table 1. Since laboratory variables did not follow a Gaussian distribution (Kolmogorov–Smirnov test), data were expressed as median and range (2.5–97.5 percentiles). The percentages of impaired results for each variable and group are also stated. ROC analysis was performed for all variables, and the results are stated in additional file 3. For neopterin, the previously calculated cut-off value of 61 nmol/L for a paediatric population displayed good specificity and sensitivity (97.2% and 81.2%, respectively). After the transformation of the continuous variables into log values, there were significant differences in the three biomarkers among the different groups (neopterin: F = 187.5, *p* < 0.0001; leukocytes: F = 105.1, *p* < 0.0001; total proteins: F = 49.4, *p* < 0.0001, ANOVA with Bonferroni correction). Regarding neopterin, the highest values were observed in the bacterial and viral meningitis groups, while the lowest were found in the acquired autoimmune disease and control groups. Considering CSF leukocytes, bacterial followed by viral meningoencephalitis displayed the highest values and showed significant differences when compared with the other groups. For CSF total proteins, the highest values were detected in the bacterial infection group, which showed significant differences when compared with all of the other groups. Finally, the acquired autoimmune group showed the highest patient age values. Details of the CSF biomarker values and significant differences among the four patient groups are stated in Table 1, Fig. 1 and additional file 4.

**Table 1:** Biochemical details for the entire patient cohort (n = 277).

Clinical group (n)	Age in years	CSF neopterin (nmol/L)	% elevated	CSF leukocytes (WBC/mm^3^)	% elevated	CSF proteins (mg/dL)	% elevated
Group A (n = 107) Viral	4.8 (3.4) (0.1–19)	238 (45–860)	96.2% (103/107)	90 (0–1821)	90.6% (97/107)	39 (15–134)	45.8% (49/107)
Group B (n = 15) Bacterial	2.7 (2.8) (0.1–9)	307 (54–1841)	93.3% (14/15)	290 (0–10,421)	80% (12/15)	135 (15–490)	86.7% (13/15)
Group C (n = 48) Immune	8.3 (5.01) (1–17)	46 (11–648)	43.7% (21/48)	9.6 (0–135)	54.1% (26/48)	36 (15–353)	41.6% (20/48)
Group D (n = 107) Controls	6.1 (5.9) (0.1–21)	23 (8–69)	1.86% (2/107)	0 (0–10)	5.6% (6/107)	19 (12–86)	10.3% (11/107)

Age is expressed as average, SD and range. Since the rest of variables did not follow a Gaussian distribution (Kolmogorov–Smirnov test), data are expressed as median and range, the latter defined as 2.5–97.5 percentiles. Percentage (%) of impaired results are also reported: > 61 nmol/L for neopterin, > 5 leukocytes/mm^3^, and > 40 mg/dL for total protein values.

### Canonical discriminant analysis

**Figure 2:**
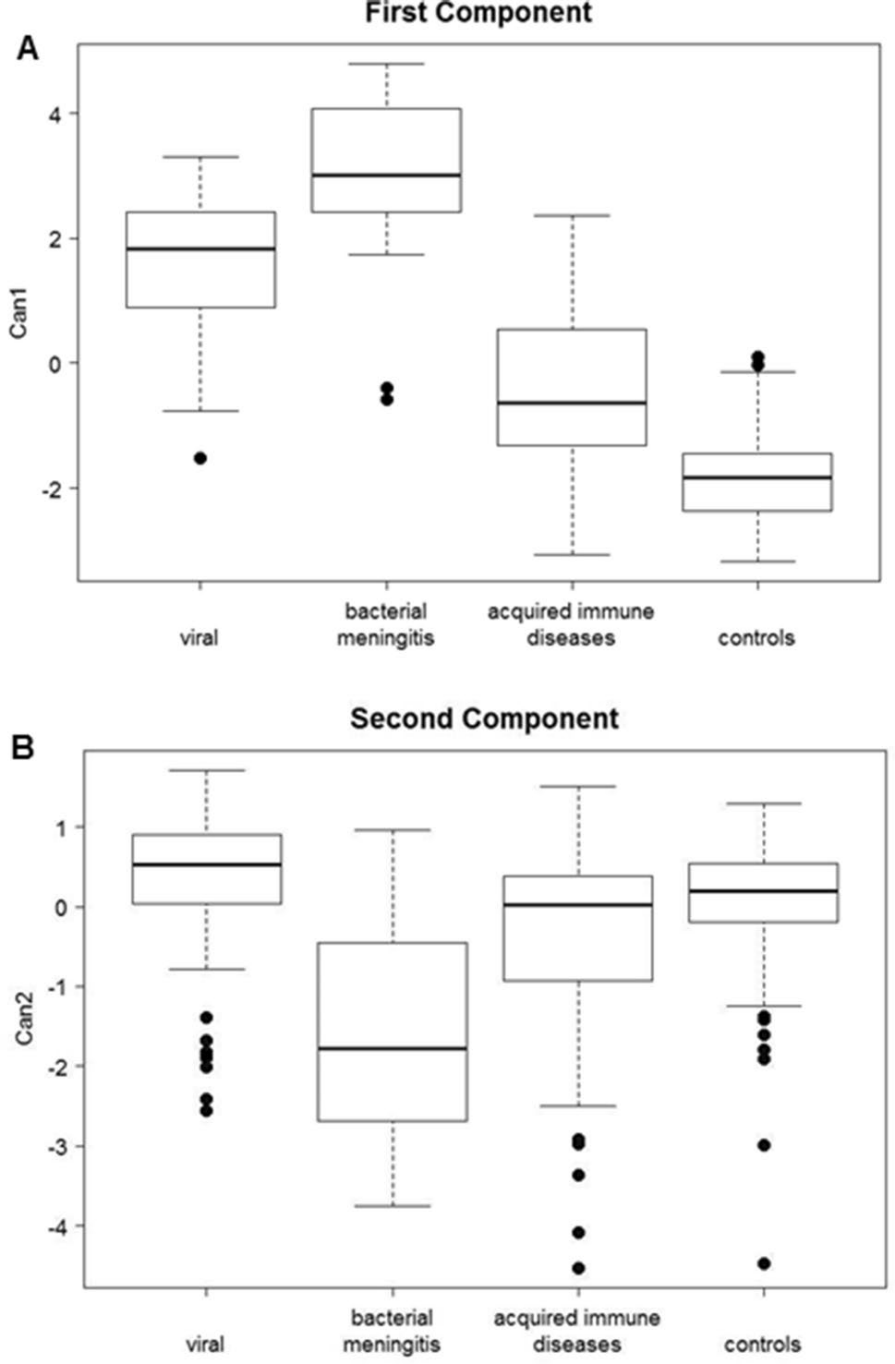
Box plot representation of the two dimensions of Can_1_ and Can_2_. (**A**) Patients from bacterial and viral infection groups had higher Can_1_ values, while the lower values corresponded to acquired autoimmune conditions and controls. (**B**) Patients with bacterial meningoencephalitis could be differentiated from the other groups since they exhibit the lowest Can_2_ values. The length of the boxes indicates the interquartile space (p25–p75); the horizontal line into the box represents the median (p50); and the circles indicate outlier values.

**Figure 3:**
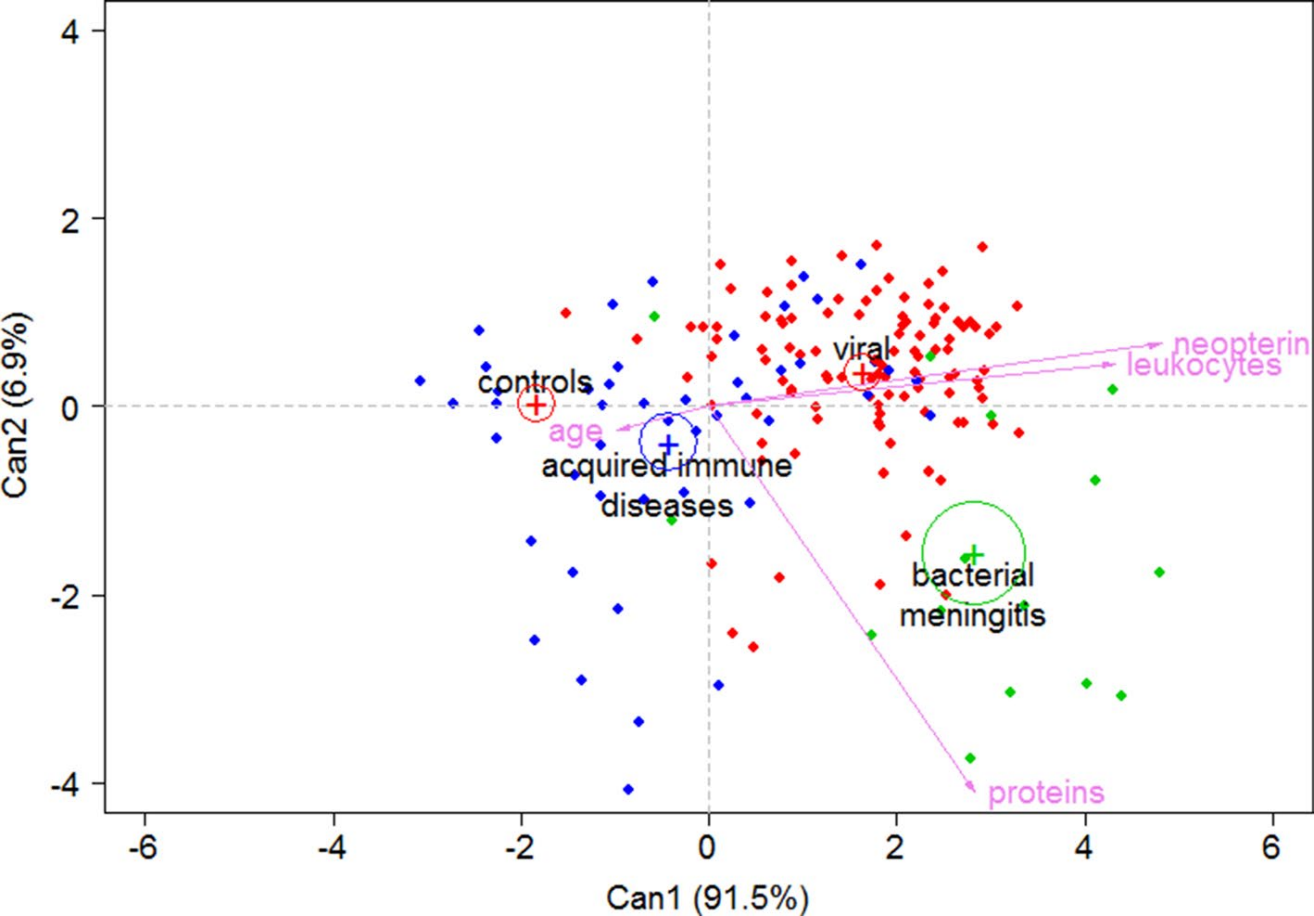
Graphical representation of each group and individual patients according to Can 1 and Can 2 dimensions. Legend: With the combination of both Can1 and Can2 dimensions, patients with acquired immune diseases and controls are separated from the other groups (negative values in Can_1_ dimension). The viral and bacterial meningitis disease groups are also differentiated in the second dimension. The points indicate each patient included in the study according to canonical discriminant analysis. The control group (green points) are positioned at the graph left part (low Can1 values caused by low values of the 3 biomarkers assessed), the viral meningitis group (red points) is positioned at the upper right part (high Can1 and Can2 values) and the bacterial meningitis group is positioned at the low right part of the graph (high Can1 and low2 values). The autoimmune acquired disease group was more randomly distributed around the central part of the graph.

We performed a canonical discriminant analysis of CSF neopterin, total proteins, leukocytes, and the age of the patients in the four groups, since the combination of the different biomarkers (Figs. 2, 3) could improve the discrimination among the different groups. Using logarithmic values for each variable except for age, we obtained three dimensions (canonical equations (Can)). Can_1_ and Can_2_ dimensions contributed to 98.4% of the total variability of the model while the third dimension was not included in the analysis (data not shown).

Can_1_ =  + 0.967 logNeopterin + 0.398 logProtein + 0.288 logLeukocytes  − 0.019 Age.

Can_2_ =  + 0.288 logNeopterin − 1.742 logProtein + 0.189 logLeukocytes + 0.031 Age.

In Can_1_, high neopterin, total proteins, and leukocyte values increased the value of this function (positive coefficient), while age had a negative coefficient. Thus, patients from the bacterial and viral infection groups had higher Can_1_ values, while the lower values corresponded to acquired autoimmune conditions and controls (Fig. 2A). Can2 had a negative coefficient for the total protein variable, while the leukocyte count, neopterin level and the age had a positive effect. Using this function, patients with bacterial meningoencephalitis could be differentiated from the other groups since they exhibit the lowest canonical values (Fig. 2B). Figure 3 shows a graphical position of the different groups and any individual patient considering the effects of the two dimensions. With the combination of both Can_1_ and Can_2_ dimensions, patients with acquired immune diseases and controls are separated from the other groups (negative values in Can_1_ dimension). The viral and bacterial meningitis disease groups are also differentiated in the second dimension. The control group is positioned in the left part, the viral meningitis group is positioned in the upper right part and the bacterial meningitis group is positioned at the low right part of the graph. The autoimmune acquired disease group was more randomly distributed around the central part of the graph.

### Leave-one-out cross and independent cohort study for validation

Table 2 shows the validation results in the four patient groups. In leave-one-out validation, the highest percentage of adequate classification was reached for the control and viral infection groups (92.5 and 89.7% of cases, respectively), followed by the bacterial (60%), and acquired autoimmune disease groups (27.1%). Regarding the validation in an independent cohort of patients (Table 2, additional file 2), 8 patients were diagnosed with acquired immune diseases, 4 with bacterial meningitis, 9 with viral meningitis and 65 were classified as controls since they did not present an immune/inflammatory event at the moment of CSF analysis. The highest percentage of adequate classification was reached for the control and viral infection groups (93.8 and 77.8% of cases, respectively), followed by the acquired autoimmune disease (75%) and bacterial meningitis groups (50%). An independent group of 9 Aicardi-Goutières syndrome patients (a severe genetic autoimmune condition) was also assessed. The model classified these patients as viral or bacterial meningitis (n = 8), and only one single case was classified as a control. This group had a median neopterin values higher than viral or bacterial meningitis patients (median 797 nmol/L; range 85–3010), while differences in leukocytes and total proteins were less remarkable (median 5, range 0–70; and median 60 mg/dL, range 15–447), representing a different group.

**Table 2:** Leave-one-out cross-validation assessment and independent cohort validation in the four patient groups.

Prediction Leave-one-out cross-validation	Viral encephalitis	Bacterial meningitis	Acquired Immune	Control group
Viral encephalitis (n = 107)	96 **89.7%**	2 1.87%	7 6.54%	2 1.87%
Bacterial meningitis (n = 15)	4 26.7%	9 **60%**	1 6.67%	1 6.67%
Acquired Immune (n = 48)	15 31.2%	0 0%	13 **27.1%**	20 41.7%
Control group (n = 107)	1 0.93%	0 0%	7 6.54%	99 **92.5%**
**Independent cohort validation**				
Viral encephalitis (n = 9)	7 **77.8%**	1 11.1%	1 11.1%	0 0%
Bacterial meningitis (n = 4)	2 50%	2 **50%**	0 0%	0 0%
Acquired immune (n = 8)	1 12.5%	0 0%	6 **75%**	1 12.5%
Control group (n = 65)	1 1.54%	0 0%	4 6.15%	60 **92.3%**
Genetic diseases (n = 9)	5 55.6%	3 33.3%	0 0%	1 11.1%

Data are expressed as number of cases and the percentage of right classifications obtained. The highest percentage of correct classification was reached for the viral encephalitis and control groups in both assessments (percentages highlighted in bold), followed by the acquired immune and bacterial meningitis groups. Patients from the group with genetic immune disease (Aicardi-Goutières), we classified as viral or bacterial meningitis and only one as a control.

## Discussion

Diagnosis workflows, prognosis, and therapeutic approaches differ depending on the aetiology of a neuroinflammatory disorder. Therefore, early and accurate classification of these diseases is important. Sometimes prompt diagnosis is not easy because there are still very limited CSF biomarkers, especially in children with inflammatory or autoimmune brain diseases^27^. However, viral and especially bacterial infections are identified by other highly effective microbiological diagnostic approaches. Leukocyte count and total protein concentration in the CSF are comprehensively studied in many neuropaediatric disorders, even though their sensitivity and specificity are not very high^7,8^. CSF neopterin values higher than 61 nmol/L are useful to discriminate between inflammatory and non-inflammatory neurological disorders, but they cannot discriminate among different neuroinflammatory aetiologies^8^. Regarding the different clinical groups studied here, the percentage of patients with increased neopterin values (> 61 nmol/L) was higher than the other biomarkers (leukocytes and total proteins) in most groups. This finding is in good agreement with the sensitivity of neopterin as a biomarker in inflammatory/immune cases, as previously reported^7,8^. The cut-off values calculated by ROC analysis for CSF protein and leukocyte values to discriminate between inflammatory and non-inflammatory neurological disorders are similar to those previously reported^28^. When ROC analysis was applied among the different clinical groups and controls, neopterin displayed the highest sensitivity and specificity when compared with proteins and leukocytes. However, the cut-off values overlapped among the clinical groups (additional file 3).

In this study, we assessed the combination of neopterin with two well-established CSF biomarkers (total protein and leukocyte values) and showed that the early classification of patients affected by neuroinflammatory disorders can be improved. It has long been argued that there is still a need to develop biomarkers in neuroimmunological diseases, particularly in the paediatric population^29^.

In the field of infectious meningitis, the combination of multiple biomarkers in the form of a “meningitis score for emergencies” has proven useful for the discrimination between aseptic and bacterial meningitis^30^. Following a similar rationale, our study aims to explore which biomarker (or combination of biomarkers) would be more useful to discriminate a wider spectrum of neuroinflammatory diseases, including viral meningoencephalitis, bacterial meningitis and acquired immune-mediated disorders.

To further classify the patients, we used a canonical discriminant analysis with three biochemical variables (neopterin, leukocytes, and total proteins), along with the age of the patients. With the combination of both dimensions, our results showed that controls and autoimmune acquired conditions could be discriminated from the other groups. Additionally, the bacterial and viral meningitis groups were discriminated as well (Fig. 3). Validation study results confirmed these observations. Sixty-one out of 65 cases identified as controls were properly classified. These were neuropaediatric patients who underwent lumbar puncture during 2019 for the diagnosis of epilepsy and other complex neurological pictures with no initial clinical suspicion of inflammatory diseases. The 4 cases that were classified as acquired autoimmune diseases (additional file 2) included a patient with vascular stroke, a patient with leukodystrophy and 2 cases with demyelinizing disease associated with other neurological signs. Either unspecific inflammatory events associated to brain damage or even a possible autoimmune aetiology would explain this classification. In any case, autoantibody analysis in these patients is advisable.

Six out of 8 acquired autoimmune disease patients were correctly classified, while 2 cases were classified as a control patient and a viral meningitis patient. The diagnosis of this group of patients is especially complex since laboratory investigations to elucidate the aetiology are not available in most laboratories, and these results may be useful for a rapid orientation for further investigations. Regarding patients with viral infections, 7 out of 9 were correctly classified and only failed in 2 cases with parvovirus and herpes virus who were classified as acquired immune disease and bacterial infection, respectively. In these cases, differences in neopterin values explained this misclassification, since the patient with herpes meningitis displayed a very high CSF neopterin value, while the case with parvovirus showed moderately increased values (additional file 2). An independent group of 9 Aicardi-Goutières syndrome patients was also assessed. The model classified these patients as viral or bacterial meningitis (n = 8), and only one single case was classified as a control. This is probably due to very high values of CSF neopterin being displayed by this group, which exerted a high influence on the final results of the Can1 dimension. From a clinical point of view, the lack of discrimination between these groups may not be relevant, because there are highly effective clinical and microbiological tests that can make the differential diagnosis between a bacterial/viral infection and an autoimmune genetic disorder^19–22^. Moreover, patients with Aicardi–Goutières syndrome have a complex and chronic neurological phenotype, which is completely different from that of bacterial or viral meningoencephalitis^16,24^. Additionally, some Aicardi–Goutières patients may present normal inflammatory biomarker results over the evolution of the disease.

We decided to join patients with both viral infections and lymphocytic aseptic meningoencephalitis, since these groups displayed similar positions in the graphical representation after a preliminary canonical discriminant analysis, and after cross-validation analysis, most of the lymphocytic aseptic meningoencephalitis patients were classified as viral infections (data not shown). This approach is in agreement with the diagnosis since in our cohort of patients with lymphocytic aseptic meningoencephalitis, bacterial and autoimmune aetiologies were ruled out with extensive microbiological and autoantibody studies. Furthermore, it has been demonstrated that the majority of patients with lymphocytic aseptic meningoencephalitis end up being diagnosed with a viral infection or remain undiagnosed^31^. Regardless, neither have a bacterial infection nor an autoimmune genetic disorder.

The biological roles of neopterin in inflammation are still a matter of debate, especially in the central nervous system. Both the protective and potential deleterious roles of neopterin in cells and its implications in the clinical outcome of patients deserve further investigations^9^.

A limitation of the study may be due to a potential bias of patient group recruitment, since other groups of diseases such as those presenting concomitant unspecific inflammatory events without a final diagnosis were not included in the study design. Most likely, due to the moderate increment in neopterin values in this group as previously reported^8^, it might be classified as acquired autoimmune diseases, as we observed in our 4 controls from the patient cohort for model validation. Another important issue is that CSF samples cannot be taken from a healthy paediatric population, since it is an aggressive procedure, and in paaediatric patients it is always done because children are sick. Although we ruled out traumatic punctures or samples with signs of central nervous system inflammation or infection in all of the CSF control samples, it is very difficult to be sure that subtle inflammatory events that can slightly increase neopterin values are not present. This fact could explain the relatively high CSF neopterin values from our paediatric population when compared with adults^32^.

## Conclusion

This study provides initial evidence of a more efficient approach to promote the timely classification of patients with viral and bacterial infections, acquired autoimmune disorders, and neuropaediatric patients with no immune diseases. Through canonical equations, we have applied a new tool that may aid in the rapid differential diagnosis of these groups of diseases. These results may guide future studies to better discriminate among neuroinflammatory diseases.

## Supplementary information

Supplementary Additional file 1.

Supplementary Additional file 2.

Supplementary Additional file 3.

Supplementary Additional file 4.

Supplementary Information 5.

## Data Availability

The datasets used and/or analysed during the current study are available from the corresponding author on reasonable request. The data generated after the cohort study for model validation are included in this published article (as supplementary file).

## Abbreviations

CSF: Cerebrospinal fluid
BH4: Tetrahydrobiopterin
GTPCH: Guanosine triphosphate cyclohydrolase I
CBA: Cell-based assay
Log: Logarithmic
Can: Canonical
