# Supplementary figures and images for: Cerebrospinal fluid neopterin as a biomarker of neuroinflammatory diseases
Source: Sci Rep. 2020 Oct 26;10:18291. doi: 10.1038/s41598-020-75500-z (PMC7588460; doi:10.1038/s41598-020-75500-z)

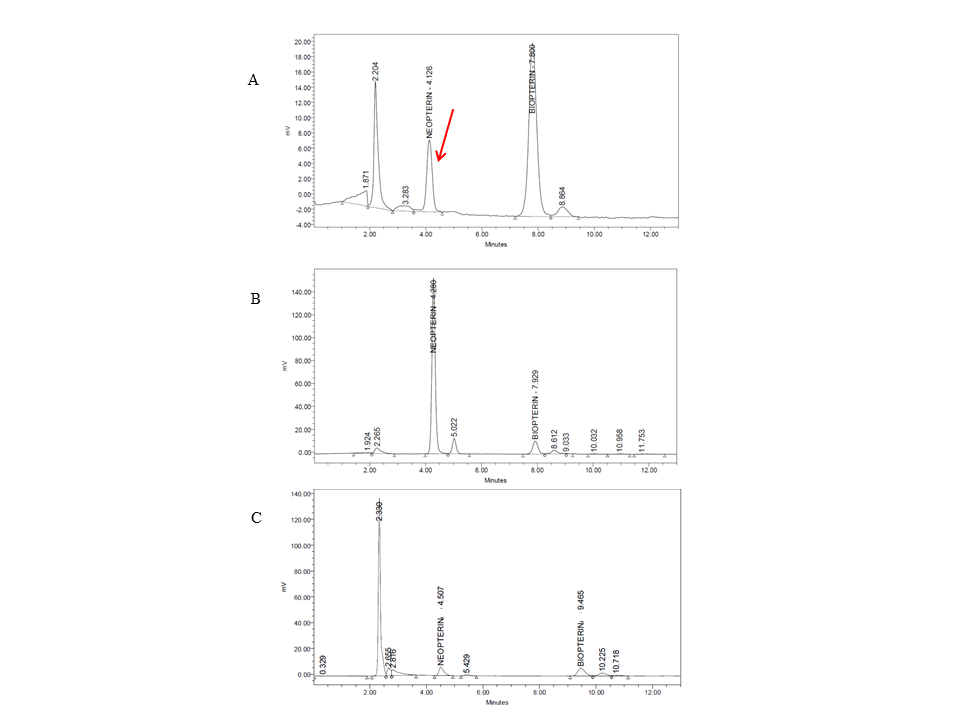

Supplement: Supplementary file 1 — Supplementary Additional file 1. [file 41598_2020_75500_MOESM1_ESM.tif]
