# Supplementary material for: Cerebrospinal fluid neopterin as a biomarker of neuroinflammatory diseases
Source: Sci Rep. 2020 Oct 26;10:18291. doi: 10.1038/s41598-020-75500-z (PMC7588460; doi:10.1038/s41598-020-75500-z)
Supplement: Supplementary file 3 — Supplementary Additional file 3. [file 41598_2020_75500_MOESM3_ESM.docx]

**Additional file 3.** ROC analysis: Optimal cut-off values for all individual markers (neopterin, leukocytes and total protein concentrations) in patients with neurinflammatory diseases vs controls. Specificity and sensitivity is expressed as %. AUC: Area under the curve. Cut-off values are exprsend as nmol/L neopterin), mg/dl (total proteins) and WBC/mm^3^ (leukocytes)

AUC Cut-off Specificity Sensitivity

All patients vs controls

Neopterin 0.916 61 97.2 81.2

Proteins 0.805 27.5 76.7 75.3

Leukocytes 0.882 6.5 94.4 78.8

Patients with viral meningoencephalitis vs controls

Neopterin 0.989 63 97.2 96.3

Proteins 0.808 27.5 75.7 76.6

Leukocytes 0.936 6.5 94.4 90.7

Patients with bacterial meningitis vs controls

Neopterin 0.993 51 93.52 100

Proteins 0.925 58.5 93.5 86.7

Leukocytes 0.892 13.3 99.1 80.0

Patients with acquired immune-mediated disorders vs controls

Neopterin 0.729 40.5 86.9 52.1

Proteins 0.936 27.5 75.7 66.7

Leukocytes 0.757 3.5 62.6 72.9

The conclusion of this ROC analysis is that the canonical approach is necessary, since either sensitivity or specificity reached for some clinical groups by analysis of individual biomarkers is low, and the cut-off value overlapped amongst the different groups. The cut-off value of 61 nmol/L previously calculated for pediatric patients displayed a good specificity and sensitivity (97.2% and 81.2% respectively).
