# Supplementary material for: Cerebrospinal fluid neopterin as a biomarker of neuroinflammatory diseases
Source: Sci Rep. 2020 Oct 26;10:18291. doi: 10.1038/s41598-020-75500-z (PMC7588460; doi:10.1038/s41598-020-75500-z)
Supplement: Supplementary file 4 — Supplementary Additional file 4. [file 41598_2020_75500_MOESM4_ESM.docx]

**Supplementary table 2**. Statistically differences among clinical groups using the ANOVA with Bonferroni correction parametric test. For neopterin, the highest values were observed for the groups of viral and bacterial meningitis. For CSF leukocytes, the groups of bacterial meningitis followed by viral meningoencephalitis showed the highest values. For CSF proteins, the highest values were detected in the bacterial meningitis group.

Groups:

A. Viral meningitis.

B. Bacterial meningitis.

C. Acquired autoimmune diseases

D. Control group

|  | **Patient´s Average**  **groups p value Confidence interval differences** | | | | | | | |
| --- | --- | --- | --- | --- | --- | --- | --- | --- |
| **Log Neopterin** | | A | B | .185 | -1.005 | .101 | -.452 |  |
|  |  |  | C | .000 | 1.083 | 1.781 | 1.432 |  |
|  |  |  | D | .000 | 2.014 | 2.565 | 2.290 |  |
|  |  | B | C | .000 | 1.290 | 2.478 | 1.884 |  |
|  |  |  | D | .000 | 2.188 | 3.296 | 2.742 |  |
|  |  | C | D | .000 | .508 | 1.207 | .857 |  |
| **Log Protein** | | A | B | .000 | -1.655 | -.802 | -1.229 |  |
|  |  |  | C | 1.000 | -.294 | .243 | -.025 |  |
|  |  |  | D | .000 | .348 | .772 | .560 |  |
|  |  | B | C | .000 | .745 | 1.661 | 1.203 |  |
|  |  |  | D | .000 | 1.362 | 2.216 | 1.789 |  |
|  |  | C | D | .000 | .316 | .855 | .586 |  |
| **Log Leukocytes** | | A | B | .453 | -1.932 | .379 | -.776 |  |
|  |  |  | C | .000 | 1.425 | 2.882 | 2.154 |  |
|  |  |  | D | .000 | 3.001 | 4.151 | 3.576 |  |
|  |  | B | C | .453 | -.379 | 1.932 | .776 |  |
|  |  |  | D | .000 | 1.690 | 4.170 | 2.930 |  |
|  |  | C | D | .000 | .692 | 2.151 | 1.422 |  |
