# Supplementary material for: Cerebrospinal fluid neopterin as a biomarker of neuroinflammatory diseases
Source: Sci Rep. 2020 Oct 26;10:18291. doi: 10.1038/s41598-020-75500-z (PMC7588460; doi:10.1038/s41598-020-75500-z)
Supplement: Supplementary file 5 — Supplementary Information 5. [file 41598_2020_75500_MOESM5_ESM.docx]

**Cerebrospinal fluid neopterin as a biomarker of neuroinflammatory diseases**

^1,2^Marta Molero-Luis, PhD, ^1,3^Didac Casas-Alba, MD, PhD, ^3^Gabriela Orellana, MD, ^1,2^Aida Ormazabal PhD, ^1,2^Cristina Sierra, PhD, ^2^Clara Oliva, BsC, ^2^Anna Valls, MD ^2^Jesus Velasco, MD ^1,4^Cristian Launes, MD, PhD, ^5^Daniel Cuadras, PhD, ^6^Belén Pérez-Dueñas, MD, PhD, ^7,8^Iolanda Jordan, MD, PhD, ^7^Francisco J. Cambra, MD, PhD, ^3,9^Juan D. Ortigoza-Escobar, MD, PhD, ^1,10,11,12^Carmen Muñoz-Almagro, MD, PhD, ^1,3^Angels Garcia-Cazorla, MD, PhD, ^13,14^Thais Armangué* MD, PhD, ^1,2,10^Rafael Artuch*, MD, PhD.

*TA and RA are co-senior authors.

1. Institut de Recerca Sant Joan de Déu, Barcelona, Spain.
2. Clinical biochemistry Department. Hospital Sant Joan de Déu, Barcelona, Spain.
3. Pediatric Neurology Department. Hospital Sant Joan de Déu, Barcelona, Spain
4. Pediatrics Department. Hospital Sant Joan de Déu, Barcelona, Spain
5. Fundació Sant Joan de Déu, Barcelona, Spain.
6. Pediatric Neurology Research Group, Hospital Vall d'Hebron - Institut de Recerca (VHIR)
7. Pediatric Intensive Care Unit. Hospital Sant Joan de Déu, Barcelona, Spain.
8. Pediatric Infectious Diseases Research Group, Institut Recerca Hospital Sant Joan de Déu, CIBERESP, Barcelona, Spain
9. Movement disorder Unit ERN-RND. Hospital Sant Joan de Deu, Barcelona, Spain
10. CIBERER-Instituto de Salud Carlos III, Barcelona, Spain.
11. CIBER de Epidemiología y Salud Pública (CIBERESP), ISCIII, Barcelona, Spain.
12. Department of Medicine, Universitat Internacional de Catalunya
13. Neuroimmunology Program, Institut d’Investigació Biomèdica August Pi i Sunyer (IDIBAPS)-Hospital Clínic, University of Barcelona, Spain.
14. Pediatric Neuroinmunology Unit, Sant Joan de Deu Children’s Hospital, University of Barcelona, Spain.

**Contact information:**

Dr. Rafael Artuch. Clinical biochemistry Department. Institut de Recerca Sant Joan de Déu. Passeig Sant Jan de Déu, 2. 08950, Esplugues de Llobregat, Barcelona, Spain.

Emil: [rartuch@hsjdbcn.org](mailto:rartuch@hsjdbcn.org). Telephone: +34932806169.
